# Advances in Understanding Human Genetic Variations That Influence Innate Immunity to Fungi

**DOI:** 10.3389/fcimb.2020.00069

**Published:** 2020-02-28

**Authors:** Richard M. Merkhofer, Bruce S. Klein

**Affiliations:** ^1^School of Medicine and Public Health, University of Wisconsin-Madison, Madison, WI, United States; ^2^Department of Pediatrics, University of Wisconsin-Madison, Madison, WI, United States; ^3^Department of Medicine, University of Wisconsin-Madison, Madison, WI, United States; ^4^Department of Microbiology and Immunology, University of Wisconsin-Madison, Madison, WI, United States

**Keywords:** genes, fungi, immunity, innate, polymorphism

## Abstract

Fungi are ubiquitous. Yet, despite our frequent exposure to commensal fungi of the normal mammalian microbiota and environmental fungi, serious, systemic fungal infections are rare in the general population. Few, if any, fungi are obligate pathogens that rely on infection of mammalian hosts to complete their lifecycle; however, many fungal species are able to cause disease under select conditions. The distinction between fungal saprophyte, commensal, and pathogen is artificial and heavily determined by the ability of an individual host's immune system to limit infection. Dramatic examples of commensal fungi acting as opportunistic pathogens are seen in hosts that are immune compromised due to congenital or acquired immune deficiency. Genetic variants that lead to immunological susceptibility to fungi have long been sought and recognized. Decreased myeloperoxidase activity in neutrophils was first reported as a mechanism for susceptibility to *Candida* infection in 1969. The ability to detect genetic variants and mutations that lead to rare or subtle susceptibilities has improved with techniques such as single nucleotide polymorphism (SNP) microarrays, whole exome sequencing (WES), and whole genome sequencing (WGS). Still, these approaches have been limited by logistical considerations and cost, and they have been applied primarily to Mendelian impairments in anti-fungal responses. For example, loss-of-function mutations in *CARD9* were discovered by studying an extended family with a history of fungal infection. While discovery of such mutations furthers the understanding of human antifungal immunity, major Mendelian susceptibility loci are unlikely to explain genetic disparities in the rate or severity of fungal infection on the population level. Recent work using unbiased techniques has revealed, for example, polygenic mechanisms contributing to candidiasis. Understanding the genetic underpinnings of susceptibility to fungal infections will be a powerful tool in the age of personalized medicine. Future application of this knowledge may enable targeted health interventions for susceptible individuals, and guide clinical decision making based on a patient's individual susceptibility profile.

## Susceptibility to Fungal Diseases

We are constantly exposed to fungi capable of causing disease. Every human is colonized with a commensal fungal mycobiome (Huffnagle and Noverr, 2013), and it is nearly impossible to eliminate the spores of saprophytic environmental fungi even from the cleanest settings (Oberle et al., 2015). It should not be surprising, then, that superficial fungal infections are among the most common infectious diseases worldwide (White et al., 2014). Yet, remarkably, most people do not regularly get fungal infections, and invasive fungal infections are rare in the general population (Bongomin et al., 2017; Li et al., 2017).

One population at high risk of fungal illness is those who are immunocompromised. This has long been recognized; for example, Acquired Immunodeficiency Syndrome (AIDS) was first reported as a case series of opportunistic fungal infections in homosexual men (Centers for Disease Control, 1981). The number of “at risk” immunocompromised people is growing largely due to secondary causes, e.g., immunosuppressive drugs used in the setting of malignancy, organ transplantation, or autoimmune disease (Yapar, 2014; Pana et al., 2017).

Primary immunodeficiencies (PIDs) continues to be a threat. PIDs are inherited defects in the immune response; depending on the affected pathway(s), PIDs have different severity, onset, and risks of infection by certain groups of organisms. Perhaps the most dramatic PID is Severe Combined Immunodeficiency (SCID), a condition where the absence of an adaptive immune response uniformly leads to overwhelming infections and death in the first few years of life in the absence of stem cell transplantation. SCID was first described in 1950 and soon recognized as heritable (Buckley, 2004).

A number of monogenic deficits in immunity engender fungal illness. For example, multiple different loss-of-function mutations in *CARD9* have been associated with autosomal recessive inheritance of susceptibility to invasive infections by fungi, including *Candida* spp., dermatophytes, and fungal plant pathogens (Vaezi et al., 2018). Understanding the immune deficits that underlie “idiopathic” fungal disease in otherwise healthy people has provided valuable insight into human antifungal immunity, and there is clinical utility to the diagnosis of these conditions (e.g., genetic counseling, antifungal prophylaxis) (Li et al., 2017). Attribution of these PIDs to biological processes has historically relied on a “candidate gene” approach, where animal models of disease and *in vitro* assays with patient cells have guided the search for “lesions” in select genes.

While monogenic, “Mendelian” susceptibility variants have revealed the pathways that are critical for control of different pathogens, they are unlikely to explain population-level patterns in risk of fungal disease. Such monogenic “lesions” in non-redundant immune pathways are overwhelmingly deleterious; they confer a substantial risk of illness, and as a result there is strong purifying selection that favors recognition and control of infection (Netea et al., 2012).

Fine variations in the immune responses that do not result in susceptibility to overwhelming infection are more likely to explain population-level trends in fungal illness. Susceptibility to mild infections may be maintained in a population because overly exuberant antimicrobial immune responses can be disadvantageous; they may cause undue tissue pathology in response to infection (Jaeger et al., 2016), or engender autoimmunity (Zhernakova et al., 2010; Ramos, 2017). Thus, a person's genetic susceptibility is the net result of many variants that each alter immune function in nearly imperceptible ways. Such variants are under substantially less selective pressure than those underlying PIDs and Mendelian susceptibility variants, and are more prone to changes in frequency due to stochastic effects like genetic drift (Netea et al., 2012).

Advances in bioinformatic approaches have made it more practical to screen a large number of donors, and to search widely in the genome for rare variants or mutations. This has facilitated the discovery of many common variants that confer a small risk of select infections. High-quality studies seeking such risk variants will involve a large number of participants that share a well-defined set of clinical parameters, plus a large number of well-matched participants serving as controls, and efforts will be made to validate functional impacts of risk variants. Validation may not always be possible due to the limitations of experimental systems, in which case animal models of illness may provide corroborating evidence to demonstrate biological feasibility. Consideration must be given to confounding factors that could introduce error. One notable confounder in the search for risk variants is ethnic/racial differences between study group and control. Thus, even a well-conducted study may have limited generalizability as it only involves participants of a given genetic background. Replicating studies with cohorts from different genetic backgrounds is valuable in beginning to understand how given risk variants may be masked by epistatic effects. Throughout this review, the ethnic/racial background of participants in a given study is reported using the demographic terms used by the authors of the study.

This review addresses genetic susceptibility to fungal infection, with an emphasis on the growing understanding of common, subtle variants that underlie population-level patterns of disease. We present “case studies” of illness caused by four groups of fungal pathogens: dermatophytes, *Candida* spp., *Aspergillus* spp., and dimorphic fungi endemic to the United States. Each section begins with an overview of relevant ecological, evolutionary, and epidemiologic features, then proceeds to human susceptibility to infection. Fungal diseases are discussed in order of global prevalence.

## Dermatophytosis

Dermatophytic fungal diseases are among the most common infectious diseases worldwide (White et al., 2014; Bongomin et al., 2017). This group includes fungi with diverse natural histories and clinical courses. Dermatophytes are a monophyletic group of ascomycetes of the genera *Trichophyton, Microsporum*, and *Epidermophyton* (White et al., 2014). Anthropophilic species are found exclusively on humans, zoophilic species are found on a number of different animal hosts, and geophilic species are found in the environment (White et al., 2014). Anthropophilic species are capable of a commensal lifestyle on human hosts, zoophilic species generally cause chronic or mild illness in humans, and geophilic species that are not adapted to live hosts are a rare cause of acute illness (White et al., 2014). Studies have identified a number of virulence factors that determine pathogenicity of dermatophytes (Gnat et al., 2019).

Dermatophyte infection can lead to illness characterized by scaly, pruritic rashes. These illnesses are often named by the location; e.g., tinea pedis for infections affecting the feet, and tinea capitis for infections affecting the scalp. Local prevalence of dermatophytic illness is reported to be over 70% in some populations, and up to one billion people are affected globally (Bongomin et al., 2017). The development of illness is dependent on environmental factors like hygiene and humidity; thus, prevalence of illness is lower in developed areas (Bongomin et al., 2017). Though dermatophytes are primary pathogens in that they cause illness in otherwise healthy people, disease is often mild and self-limiting, and exposure to and colonization with these fungi without associated disease is well-described (Abdel-Rahman et al., 2006; Gnat et al., 2019).

### Superficial Dermatophytosis

Dermatophyte infection was the subject of the first genome wide association study (GWAS) of fungal infection. Abdel-Rahman et al. (2006) studied 446 predominantly African-American children over 2 years in an urban daycare center, documenting symptoms of tinea capitis and carriage of the most commonly associated dermatophyte (*T. tonsurans*). They found that donors were either exclusive carriers of one strain, predominant carriers of one strain with occasional other strains recorded, or “random” carriers who did not appear to have a predilection for one strain (Abdel-Rahman et al., 2006). Intriguingly, “random” carriers had the fewest symptomatic infections (Abdel-Rahman et al., 2006). Abdel-Rahman and Preuett (2012) then did whole genome genotyping on 20 children who carried *T. tonsurans* >90% of the time, and 20 children who carried the fungus <10% of the time. The authors identified 21 genes whose genotype was associated with carriage of the fungus, though they did not study whether this was correlated with symptoms of tinea capitis. Genes uncovered in this GWAS were associated with various different functions, including leukocyte function, remodeling of extracellular matrix, wound repair, and cutaneous permeability (Abdel-Rahman and Preuett, 2012). Genotyping an additional 115 children, the authors found that genotype at just 8 of these genes accounted for about 60% of the variance in carriage rate (Abdel-Rahman and Preuett, 2012).

Few candidate gene studies have been carried out with patients suffering from superficial dermatophytosis. *CLEC7A-Y238X*, an early stop codon variant that impacts recognition of fungal β-glucan by the receptor Dectin-1, was reported in a Dutch family where all were affected by onychomycosis (Ferwerda et al., 2009). Subsequent studies of *CLEC7A-Y238X* have described more subtle clinical phenotypes (discussed below), and have not discussed an association with superficial dermatophytosis. Other studies of onychomycosis propose a role for variation at the Human Leukocyte Antigen (HLA) complex, which is involved with presentation of extracellular antigens to T cells and initiation of adaptive responses (Sadahiro et al., 2004; Asz-Sigall et al., 2010; Garcia-Romero et al., 2012; Carrillo-Melendrez et al., 2016). The keratinized nail is sometimes thought to be out of reach of the immune system (Gnat et al., 2019), but these studies suggest a role for both innate and adaptive immune responses to onychomycosis.

### Chronic and Deep Dermatophytosis

Early observations of disparities between those with dermatophyte illness and those at risk led to the first studies proposing a genetic contribution to dermatophytosis. Tinea imbricata, chronic dermatophytosis with a characteristic pattern of skin lesions caused by infection with *T. concentricum*, is common in some tropical areas (Ravine et al., 1980). The rate of tinea imbricata differs between people of different racial backgrounds despite similar exposure risk (Ravine et al., 1980). Illness begins early in life, and either resolves spontaneously or becomes chronic (Ravine et al., 1980). One study of 228 families in Papua New Guinea concluded that risk of persistent tinea imbricata is likely autosomal recessive (Ravine et al., 1980). A smaller study of a polygamous Mexican family found an autosomal dominant pattern of infection (Bonifaz et al., 2004). The differences between these reports may be due to the small number of people studied, or reflect a different underlying genetic determinant of susceptibility.

Immune function in patients with chronic widespread dermatophytosis (CWD) due to *T. rubrum* is characterized by selective functional deficits. Phagocytes from immunocompetent patients with *T. rubrum* CWD were less effective at killing *T. rubrum*, and produced less hydrogen peroxide and pro-inflammatory cytokines in response to the fungus, compared to cells from healthy donors (de Sousa Mda et al., 2015). The same deficits were not noted when comparing cells stimulated with maximal stimuli (e.g., LPS), or in a cohort of patients with acute tinea pedis (de Sousa Mda et al., 2015). Although genetic studies have not been done on patients with CWD, it is noteworthy that several of these functional deficits are also seen in patients with CARD9 deficiency (Drummond et al., 2015; Liang et al., 2015). CARD9 is the intracellular adaptor for several pattern recognition receptors (PRRs), including several C-type lectin receptors (CLRs) that are critical for recognition of fungi (Zhong et al., 2018). Loss-of-function mutations in *CARD9* are the only known genetic etiology underlying deep dermatophytosis, where dermatophytes invade through skin and often disseminate (Lanternier et al., 2013; Gnat et al., 2019). The immune deficits that render patients with CARD9 deficiency susceptible to fungal infection are discussed at length below, in the section on “idiopathic” infections by *Candida* spp.

### Summary

Dermatophytosis is one of the most common infectious diseases globally, often presenting as self-limiting superficial infections. Environmental factors strongly influence the development of disease. Still, carriage of dermatophytic fungi is correlated with illness, and this is influenced strongly by a small number of genes involved in leukocyte function and other processes (Abdel-Rahman and Preuett, 2012). Multiple studies have proposed a role for host genetics in the development of illness, including observations of possible Mendelian inheritance patterns for chronic dermatophytosis (Ravine et al., 1980; Bonifaz et al., 2004). Candidate gene studies implicate fungal recognition, especially signaling through the adaptor CARD9, as a critical component in controlling dermatophytosis (Lanternier et al., 2013).

## Candidiasis

*Candida* spp. are responsible for an estimated 138 million cases per year of recurrent vulvovaginal candidiasis (RVVC), in excess of 3 million cases per year of mucosal candidiasis (including oropharyngeal and esophageal), and about 750 thousand cases per year of invasive candidiasis and candidemia (Bongomin et al., 2017; Denning et al., 2018). *Candida* spp. are the fourth leading pathogen, and most common fungal pathogen causing healthcare-associated infections (Magill et al., 2018). Candidemia is a feared complication of immunosuppression, with case mortality rates of over 20% (Pagano et al., 2017). Still, the most prevalent clinical illness caused by *Candida* spp. is, by far, primary infections in immunocompetent women (Sobel, 2007; Bongomin et al., 2017; Denning et al., 2018).

Colonization with *Candida* spp. occurs within the first month of life (Ward et al., 2018), and *Candida* spp. are known to be commensal with humans (Soll et al., 1991; Huffnagle and Noverr, 2013; Neville et al., 2015; Nash et al., 2017). There is evidence that colonization with *Candida* spp. is important in the development of antifungal immunity, conferring protection against future fungal illness (Shao et al., 2019). Yet genetic typing has found that the *Candida* strains that cause disease originate from the healthy mycobiome (Odds et al., 1989; Gouba and Drancourt, 2015). Fungal biology plays a role in the disease process; the transition from the “commensal” yeast morphology to a “pathogenic” filamentous morphology is a well-studied virulence factor for *C. albicans* (Cheng et al., 2011; Cassone and Cauda, 2012; Pais et al., 2019).

Though *C. albicans* has long been the most common agent of candidiasis, the number of infections attributed to non-*albicans Candida* spp. (NAC) is increasing (Bongomin et al., 2017). This is concerning since the NAC generally have a higher level of resistance to clinically important antifungals (Bongomin et al., 2017). *Candida* is not a monophyletic genus; *C. glabrata*, the most common etiological agent of NAC infections, is more closely related to *Saccharomyces cerevisiae* than *C. albicans* (Fitzpatrick et al., 2006). One notable difference is that *C. glabrata* does not undergo the yeast-to-pseudohyphae transition that is important for pathogenicity of *C. albicans* (Pais et al., 2019).

### Vulvovaginal Candidiasis (VVC)

VVC affects about 75% of women at least once in their life (Sobel, 2007; Denning et al., 2018). Estimating the prevalence of VVC is complicated by a lack of diagnostic workup and reporting. Relying on self-reported data carries a risk of including myriad other conditions that may be mistaken for VVC. After taking this into account, Denning *et al*. estimated that about 138 million women per year are affected by RVVC (defined as four or more cases of VVC in 1 year) (Denning et al., 2018).

While most cases of RVVC are not attributable to a known comorbidity, many conditions are known to increase risk (Sobel, 2007; Denning et al., 2018). These include diabetes, cystic fibrosis, antibiotic use, pregnancy, and hormone replacement therapy (Sobel, 2007; Denning et al., 2018). Intriguingly, HIV infection and immunodeficiency do not confer risk of VVC; this, along with corroborating studies of a murine model of VVC, suggests that adaptive immunity is dispensable for prevention of VVC (Verma et al., 2017; Peters et al., 2019). Some features of the vaginal microbiome may confer resistance to VVC (Zangl et al., 2019). There are reported differences in the vaginal microbiomes of women of different ethnicities, even when donors live in the same area (Ravel et al., 2011). This may account for some of the inconsistencies in reported associations between select variants and risk of VVC when studies are carried out in genetically distinct populations.

An increasing number of genetic studies of susceptibility to RVVC support a critical role for innate immunity. Mannose-binding lectin (MBL, coded by the gene *MBL2*) is a soluble CLR that activates the complement cascade, facilitating opsonophagocytosis of fungi (Brouwer et al., 2008). A meta-analysis of five candidate gene studies found that a common loss-of-function variant *MBL2* allele B confers risk of RVVC when compared to the wild-type *MBL2* allele A (Nedovic et al., 2014). The risk of RVVC with *MBL2* genotype B,B was substantial (*MBL2* genotype B,B vs. A,A; odds ratio = 12.68, 95% confidence interval = 3.74–42.92) (Nedovic et al., 2014). This meta-analysis included studies in European and East Asian (Chinese) donors, and findings have since been replicated by studying *MBL2* promoter variants in South Asian (Indian) donors (Kalia et al., 2017). Interestingly, studies with European (Italian) (Milanese et al., 2008) and Mexican (Velazquez-Hernandez et al., 2017) donors have failed to find an association between variation at *MBL2* and risk of RVVC. This could reflect either slight differences in donor characteristics, or unappreciated epistatic effects masking the role of *MBL2* in these populations.

Risk variants have been identified in other receptors involved in the innate recognition of *Candida* spp. Recently, a WES approach in two independent cohorts of Northern and Southern Europeans identified variants in *SIGLEC15* associated with RVVC (Jaeger et al., 2019). The authors go on to validate that SIGLEC15, a lectin that binds sialic acid-containing structures, binds *Candida* spp. and is upregulated by immune cells upon exposure to *Candida* (Jaeger et al., 2019). They further demonstrate that risk variants lead to an altered cytokine profile after stimulation of immune cells with heat-killed *C. albicans* yeast, and that SIGLEC15 silencing leads to an increased inflammatory response and fungal burden in a murine model of vaginal candidiasis (Jaeger et al., 2019). Recognition of β glucans in the yeast cell wall may be important in RVVC. A coding variant in *TLR2* is associated with RVVC (Rosentul et al., 2014a), and multiple studies have proposed a role for Dectin-1. *CLEC7A-Y238X*, a variant that decreases the ability of Dectin-1 to recognize to β glucan, was found to confer risk in studies of Dutch (Ferwerda et al., 2009) and Caucasian (De Luca et al., 2013) women, but not in Western-European (Rosentul et al., 2014a), Turkish (Usluogullari et al., 2014), or Iranian (Zahedi et al., 2016) women. A study of Indian women found other *CLEC7A* variants to be associated with risk of RVVC (Kalia et al., 2018). Studies have failed to find associations between RVVC and polymorphisms in other PRRs (*TLR1, TLR4, NOD2*) or *CARD9* (Morre et al., 2002; van der Graaf et al., 2006a; Rosentul et al., 2014a).

There are conflicting reports on the role of the inflammasome and IL-1β signaling in the risk of RVVC, perhaps suggesting alternative mechanisms for susceptibility to RVVC. *NLRP3* encodes one component of the inflammasome, which cleaves and activates the pro-inflammatory cytokines IL-1β and IL-18. Intron 4 of *NLRP3* contains a variable number tandem repeats (VNTR). Decreased activity of *NLRP3*, associated with *NLRP3* allele 7, is associated with RVVC (Lev-Sagie et al., 2009). Donor cells stimulated with *C. albicans* produce less IL-1β when carrying *NLRP3* genotype 7,7 vs. 12,12 (Lev-Sagie et al., 2009). Decreased *NLRP3* activity also confers susceptibility to VVC in a murine model (Bruno et al., 2015). Interestingly, another study found the opposite association between *NLRP3* activity and RVVC. Jaeger et al. (2016) reported that the hyper-active *NLRP3* genotype 12,9 is associated with RVVC and the *NLRP3* genotype 7,7 is not associated (*p* = 0.06). In support of their reported association, the authors noted increased IL-1β production in response to *C. albicans* for *NLRP3* genotype 12,9 vs. 12,12 (Jaeger et al., 2016). Both genetic studies of *NLRP3* involved several hundred participants of white and Western-European background, respectively (Lev-Sagie et al., 2009; Jaeger et al., 2016). Indeed, both models of susceptibility are compatible; it may be that both over- and under-activity of *NLRP3* engender symptomatic infection depending on the activity of other components of the immune response. Interestingly, infection is not a prominent feature of the spectrum of conditions caused by gain-of-function variants in *NLRP3* (Gupta et al., 2019), suggesting that overactivity of *NLRP3* is likely not sufficient to predispose an individual to RVVC. While properly calibrated host responses limit pathogenesis, both too much and too little inflammation can lead to disease (Robinson and Huppler, 2017).

A third avenue of research has implicated variants affecting cellular immunity in risk of RVVC. De Luca *et al*. described variants in *IL22A* and *IDO1* significantly associated with RVVC (and not VVC) in Caucasian women (De Luca et al., 2013). They demonstrated that risk variants were associated with decreased production of IL-22 and IDO1, and downstream deficits in calprotectin and kynurenine levels, respectively (De Luca et al., 2013). A study of Latvian women found that a variant in the *IL4* promoter was associated with higher levels of IL-4 in vaginal lavage and elevated risk of RVVC (Babula et al., 2005). Considered together, these reports suggest that IL-22-producing cells, including Th17 cells, are important in RVVC and that responses nurtured by IL-4, including Th2 cells, confer risk. Interestingly, though *IL17A* and *IL22* are highly up-regulated during infection in a murine model of VVC, there was no difference in fungal burden or neutrophil responses between *IL22*^−/−^ mice and wildtype (Peters et al., 2019). Likewise, lack of CD4+ T cells in humans, for example during AIDS, does not confer risk of RVVC.

A final mechanism that may confer risk of RVVC involves glycosylation of the vaginal mucosa. Three small studies have addressed the relationship between Lewis antigen secretor status and risk of RVVC in humans (Hilton et al., 1995; Chaim et al., 1997; Kulkarni and Venkatesh, 2004). Results from the earliest study are difficult to interpret in light of potential error due to a small number of African American donors that could account for observed differences (Hilton et al., 1995). The other two studies, however, found that non-secretor status was a risk factor for RVVC in white women (Chaim et al., 1997) and VVC in Indian women (Kulkarni and Venkatesh, 2004). Compellingly, *FUT2*^−/−^ mice, a model for non-secretors, are more susceptible than wild type controls to *C. albicans* vaginitis (Hurd and Domino, 2004; Domino et al., 2009). Underlying mechanisms for this susceptibility have not been studied. However, a growing body of evidence has related *FUT2* activity and downstream mucosal fucosylation to altered composition of the gut microbiome, risk of bacterial and viral infections, and several autoimmune conditions (Kononova, 2017). In addition to differences that exist at steady state, there is evidence linking IL-22 and *FUT2* during infection. In a mouse model of opportunistic bacterial infection, signaling through IL-22RA1 was required for Fut2 enzyme activity and the resultant fucosylation (Pham et al., 2014). Direct replacement of 2′-fucosyllactose, the product of Fut2, restrained the intestinal infection phenotype in *IL22RA1*^−/−^ mice (Pham et al., 2014).

### Opportunistic Mucosal Infections by *Candida* spp.

Oropharyngeal candidiasis (OPC) and esophageal candidiasis (OEC) are closely associated with the immunocompromised state. Together, these conditions affect about 5% of HIV-positive patients on antiretroviral therapy and 20% of those with low T cell counts (Bongomin et al., 2017). Infection with HIV may engender candidiasis by targeting and depleting *Candida*-specific CD4+ T cells (Liu et al., 2016) and otherwise impacting host responses in such a way that selects for fungal virulence (Cassone and Cauda, 2012). Another risk factor for oral candidiasis is IL-17 blockade used to treat inflammatory diseases (Nash et al., 2018); this is an interesting corollary to the pathways implicated in candidate gene studies of chronic mucocutaneous candidiasis (CMC), as discussed in the section on “idiopathic” infections by *Candida* spp. Yet, the population “at risk” is much greater than the number affected, implying a possible role for genetic susceptibility.

Studies in animal models of OPC have demonstrated that CD4+ Th17 cells are involved in the control of infection (Hernandez-Santos et al., 2013). Compensatory CD8+ Tc17 cells and innate IL-17-producing cells confer protection in the context of CD4-deficiency (Hernandez-Santos et al., 2013) or loss of pathways required for Th17 responses (e.g., *CARD9*^−/−^ mice) (Bishu et al., 2014). Type 3 innate lymphoid cells cells (ILC3), natural Th17 cells, and γδT cells are innate sources of IL-17 in this OPC model. Other models suggest a central role for IL-22 signaling in protection against OPC (Goupil et al., 2014), an interesting corollary to observed susceptibilities to RVVC in humans (discussed above). Still, multiple studies have demonstrated that immune responses to *Candida* spp. at the oral and vaginal mucosas are fundamentally different (Verma et al., 2017; Gao et al., 2019), which may account for some of the differences in the epidemiology of OPC and RVVC.

Attempts to identify human genetic variants associated with opportunistic mucosal candidiasis have yet to report significant risk variants. Candidate gene studies comparing HIV-positive patients who did and did not develop OPC have failed to find significant risks of variants in genes involved in fungal recognition [e.g., *CLEC7A* (Dectin-1), *TLR2, TLR4, TIRAP, CARD9*], cytokine production (*CASP12*), and autophagy (*ATG16L1* and *IRGM*) (Plantinga et al., 2010; Rosentul et al., 2011b, 2014b).

### Opportunistic Invasive Infections due to *Candida* spp.

Bloodstream infection with *Candida* spp. (candidemia) and disseminated candidiasis are feared complications of immunosuppression. Mortality rates of over 20% have been reported (Pagano et al., 2017), despite improvements in prevention and treatment (Yapar, 2014). While several non-genetic risk factors are known (Yapar, 2014), extensive research has addressed the possibility of genetic susceptibility to candidemia and disseminated candidiasis.

Multiple studies have addressed the potential for recognition of *Candida* ligands to impact risk of invasive disease. Recent reports have detailed a mechanism whereby decreased Dectin-1 signaling confers risk of candidemia. CD82 is involved in the clustering of Dectin-1 receptors, and the efficient activation of intracellular signaling upon ligand binding (Tam et al., 2019). Variants in *CD82* are associated with both candidemia risk and decreased cytokine production upon stimulation with fungal ligands (Tam et al., 2019). It is less clear if there is a role for variants in *CLEC7A*, the gene encoding Dectin-1. A role for *CLEC7A-Y238X* was proposed after reports that this variant is associated with increased colonization by *Candida* spp. (Plantinga et al., 2009). However, *CLEC7A-Y238X* is not associated with risk of invasive infection in studies of patients with candidemia (Plantinga et al., 2009; Rosentul et al., 2011a). Vav proteins are involved in signal transduction following CLR recognition of fungal PAMPs. Roth et al. (2016) report an association between rate of candidemia in a European cohort and a block of variants near Vav proteins, which they further support by demonstrating a critical role for Vav1/2/3 in the murine response to *C. albicans* and experimental candidemia.

TLRs are also involved in recognition of *Candida* spp., and variants may confer risk of candidemia. A large study identified three risk variants in *TLR1*, although these variants were only significant in white donors (Plantinga et al., 2012). The authors validated the functional impact of these variants, and proposed a mechanism for susceptibility involving recognition of *Candida* by TLR1/TLR2 heterodimers (Plantinga et al., 2012). This study did not identify risk variants in *TLR2* or *TLR4* (Plantinga et al., 2012), however, earlier studies did report risk variants in these genes (van der Graaf et al., 2006b; Woehrle et al., 2008); all three studies were carried out in European-ancestry donors. Studies have failed to find associations with variants in other PRRs (*TLR6, TLR9, MBL2, NOD2*), TLR adaptors (*MyD88, TIRAP*), and FCγRs (*FCGR2A, FCGR3A, FCGR3B*) (Choi et al., 2005; van der Graaf et al., 2006a; Aydemir et al., 2011; Plantinga et al., 2012). Studies in the same cohorts have failed to find risk or protective variants in genes associated with autophagy (*ATG16L1, IRGM*), generation of reactive oxygen species (*CYBA*), or chitin breakdown (*CHIT1*) (Choi et al., 2005; Rosentul et al., 2014b).

While risk variants impacting recognition of fungi are broadly associated with candidemia, variants impacting phagocyte function have been more closely related to disseminated candidiasis following candidemia. Susceptibility variants have been identified in *CXCR1*, which is involved with neutrophil killing of yeast (Swamydas et al., 2016), and *CX3CR1*, which plays a role in macrophage chemotaxis and survival (McDermott et al., 2003; Lionakis et al., 2013; Collar et al., 2018). These studies have been done in European-ancestry populations; *CX3CR1-M280*, the risk variant, is not associated with invasive candidiasis in African American donors (Lionakis et al., 2013), or RVVC in European donors (Break et al., 2015). A mouse model recapitulated the role of CX3CR1 in decreasing macrophage survival, thereby engendering susceptibility to invasive infection but not mucosal candidiasis (Lionakis et al., 2013; Break et al., 2015). Interestingly, *CX3CR1-M280* was first recognized as a protective variant for cardiovascular disease (McDermott et al., 2003). The diverse findings concerning *CX3CR1-M280* provide a good example of how susceptibility variants may be highly context specific.

Cell-mediated immunity may play a role in chronic disseminated candidiasis (CDC) in the context of neutropenia. Both a protective haplotype and a risk haplotype were identified in *IL4* in neutropenic patients (Choi et al., 2003). The protective haplotype was associated with decreased IL-4 production, suggesting that Th2 responses are deleterious in CDC (Choi et al., 2003). Other polymorphisms that impact cytokine production are associated with persistent candidemia, although not studied in the context of neutropenia. Two polymorphisms, one in *IL10* and one in *IL12B*, are associated with persistent candidemia, but not candidemia in general (Johnson et al., 2012). The authors report functional effects from these risk alleles: increased IL-10 production and decreased IFN-γ production, respectively (Johnson et al., 2012). This suggests a detrimental role of Treg cells and a beneficial role of type 1 T cell responses in limiting the duration of candidemia. Interestingly, there was no significant association between genotype at *IL10* and CDC in neutropenic patients, although both studies were carried out in European ancestry populations (Choi et al., 2003; Johnson et al., 2012). Studies have failed to find significant associations with CDC or candidemia for several other cytokines (*IFNG, IL1B, IL8, IL12A, IL18, TGFB1, TNF*), a cytokine receptor (*IL12RB1*), and a gene involved in cytokine processing (*CASP12*) (Choi et al., 2003; Johnson et al., 2012; Rosentul et al., 2012).

Unbiased studies that sought to overcome the limitations of candidate gene studies have implicated viral recognition and response pathways in candidemia. One unbiased approach is to identify novel candidate genes by using a hypothesis-generating technique such as RNAseq, then applying these findings to the analysis of a patient cohort. This workflow was used to identify a linked block of variants at the gene encoding RIG-I-like receptor (RLR) *MDA5* in association with candidemia (Jaeger et al., 2015). Another study using a similar approach implicated type I interferon signaling in the response to *Candida* spp. (Smeekens et al., 2013). The authors then studied signaling downstream of type I interferon, and reported that *STAT1* polymorphisms are associated with candidemia (Smeekens et al., 2013).

Another unbiased strategy is to study patient genomes directly with a GWAS. A GWAS with 217 European-ancestry candidemia patients reported candidate risk variants near the genes encoding *CD58, TAGAP*, and the *LCD4A*-*C1orf68* locus (Kumar et al., 2014). The authors note that linked variants at the *CD58* locus fall near several non-coding RNAs, and found that genotype at *CD58* correlated with cytokine production by macrophages in response to *Candida* but not LPS (Kumar et al., 2014). *TAGAP* contributes to T cell trafficking in the thymus and negative selection (Duke-Cohan et al., 2018). All three candidate loci have been associated with autoimmunity (Kumar et al., 2014). This GWAS dataset has been used in two further studies. Using a new approach to analyze the data, Matzaraki et al. (2017) identified 18 candidate risk variants and 31 candidate susceptibility genes. The authors noted that 9 of 31 candidate genes are involved with the complement system or coagulation, and validated a functional variant in *MAP3K8* (Matzaraki et al., 2017). Finally, Li and Oosting *et al*. found that variants at Golgi membrane protein 1 (*GOLM1*) are cytokine quantitative trait loci (cQTL) for IL-6 (Li et al., 2016); these cQTL are associated with risk of candidemia and lower levels of IL-6 in candidemia patient serum (Li et al., 2016).

### “Idiopathic” infections by Candida spp.

*Candida* spp. are a rare cause of serious or invasive fungal infections in otherwise healthy people. People suffering from such “idiopathic” infections have been the subject of extensive research revealing genetic deficits in immunity (Li et al., 2017). As reviewed above, these deficits are understood to be the result of rare, monogenic polymorphisms or mutations that are overwhelmingly deleterious, and thus under strong negative selection (Netea et al., 2012; Li et al., 2017). These conditions provide an invaluable glimpse into mechanisms of antifungal immunity. The clinical manifestation of a given immune deficit illustrates areas of antifungal immunity that are non-redundant (Netea et al., 2012). Many monogenic conditions result in Chronic Mucocutaneous Candidiasis (CMC), which involves recurrent and persistent *Candida* spp. infections of the mucosa and skin (Li et al., 2017). Others result in invasive candidiasis. This topic has been the subject of recent excellent reviews, and the treatment here will thus be brief (Li et al., 2017; Lionakis and Levitz, 2018).

SCID patients, who lack an adaptive immune system, develop mucosal infection and diarrhea from *Candida* spp., in addition to multiple other infections. SCID occurs in about 1/65,000 live births (Amatuni et al., 2019), and can result from loss-of-function mutations and variants that affect the development, proliferation, or survival of T cells and/or B cells (Buckley, 2004; Heimall et al., 2017; Lionakis and Levitz, 2018; Aluri et al., 2019). Increasingly, SCID patients are being recognized by newborn screening (before infection) and are treated with stem cell transplant, which will likely improve outcomes (Heimall et al., 2017). Even with prompt diagnosis from newborn screening or family history, *Candida* spp. cause infection in 8% of patients with SCID in the first few months of life, prior to transplant (Heimall et al., 2017). Untreated, SCID is associated with OPC in ~20% of patients and chronic diarrhea, which may be caused by *C. albicans* (Aluri et al., 2019; Parvaneh et al., 2019). Interestingly, patients with untreated SCID are reported to have *C. parapsilosis* as a commensal without apparent symptoms, and to have a higher frequency than healthy controls of *C. parapsilosis* culturable from feces (Taylor et al., 1985).

Patients with severely decreased Th17 cell counts suffer from CMC, even in the absence of more global T cell or B cell deficits (Li et al., 2017; Lionakis and Levitz, 2018). Interestingly, PIDs that selectively impact Th17 cells and IL-17 signaling are associated with fewer opportunistic bacterial infections (Li et al., 2017; Lionakis and Levitz, 2018). Patients with Hyper-IgE Syndrome (HIES), also known as Job's syndrome, have impaired STAT3 signaling and diminished Th17 cell responses, and are vulnerable to CMC (Ma et al., 2008; Milner et al., 2008; Zhang et al., 2018). While STAT3 is critical in signal transduction for several cytokines, it appears that the critical deficit that confers susceptibility to CMC is lack of IL-17-producing cells *per se* (Ma et al., 2008; Hillmer et al., 2016; Zhang et al., 2018). This is illustrated by the clinical presentations of patients with genetic deficiency in other cytokine-signaling components. Loss-of-function mutations in *IL6ST*, which codes a critical subunit of the receptors for IL-6 and multiple other cytokines that signal through STAT3, result in elevated IgE and susceptibility to infections, but IL-17 responses are relatively intact and CMC is absent (Schwerd et al., 2017; Shahin et al., 2019). Deficiency in TYK2, an intracellular adaptor for multiple cytokine receptors that signal via STAT3, is associated with multiple susceptibilities, but normal Th17 responses and, in most cases, no apparent increased risk of candidiasis (Kreins et al., 2015). However, mutations that lead to decreased numbers of IL-17-producing cells are also associated with CMC susceptibility; these include gain-of-function mutations affecting STAT1 (Zheng et al., 2015; Eren Akarcan et al., 2017; Mogensen, 2018) and loss-of-function mutations affecting RORγ/RORγT (Okada et al., 2015).

Impaired IL-17 signaling due to mutations in cytokines and cytokine receptors is also associated with susceptibility to CMC. The six members of the IL-17 cytokine family (IL-17A through IL-17F) are recognized by dimeric receptors composed of the five receptor subunits in the IL-17R family (IL-17RA through IL-17RE), with an intracellular adaptor (ACT1) (Monin and Gaffen, 2018). Loss-of-function mutations conferring susceptibility to CMC have been reported in *IL17F, IL17RA, IL17RC*, and *ACT1* (Li et al., 2017; Monin and Gaffen, 2018). An interesting corollary is the high degree of CMC seen patients with Autoimmune Polyendocrine Syndrome Type 1 (APS-1), who produce autoantibodies that neutralize IL-17 cytokines due to mutations in the autoimmune regulator gene (*AIRE*) (Humbert et al., 2018).

Mutations affecting neutrophil function confer susceptibility to invasive candidiasis, in contrast to the selective susceptibility to mucosal candidiasis associated with loss of adaptive immunity and IL-17 signaling. Patients with Chronic Granulomatous Disease (CGD) suffer from invasive fungal disease due primarily to filamentous fungi, although invasive candidiasis is also reported (Wolach et al., 2017; Kanariou et al., 2018; Lionakis and Levitz, 2018). CGD results from mutations causing defective NADPH oxidase, which abrogate the oxidative burst required for phagocytes to kill microorganisms (Wolach et al., 2017; Lionakis and Levitz, 2018).

A relatively common genetic condition that predisposes patients to invasive candidiasis is myeloperoxidase (MPO) deficiency. MPO deficient cells are unable to generate HOCl, although generation of an oxidative burst is unaffected (Klebanoff et al., 2013). Neutrophils from patients with MPO deficiency display delayed killing, unlike those from patients with CGD, which fail to kill microbes at all (Klebanoff et al., 2013). Patients with MPO deficiency are narrowly susceptible to *Candida* spp.; still, only a small fraction of patients ever develop invasive candidiasis (Klebanoff et al., 2013; Lionakis and Levitz, 2018). This may indicate that early killing of phagocytosed pathogens is critical for control of *Candida* spp., but only after other defenses have been overcome (e.g., barrier integrity) (Klebanoff et al., 2013).

Loss-of-function mutations affecting CARD9 are unique in their association with susceptibility to both CMC and invasive candidiasis (Lionakis and Levitz, 2018). CARD9 deficiency abrogates fungal recognition by CLRs and downstream neutrophil responses, with little or no impact on bacterial recognition (Drewniak et al., 2013). There are conflicting reports on whether Th17 responses are impacted by loss of CARD9 in humans (Drewniak et al., 2013; Drummond et al., 2015; Vaezi et al., 2018). Over 40% of patients with CARD9 deficiency present with infections by *Candida* spp. (Vaezi et al., 2018); a large portion of those patients present with *Candida* spp. infecting their central nervous system (CNS) (Lanternier et al., 2015; Vaezi et al., 2018). Information from case reports of CARD9 deficient patients, plus studies of CNS *C. albicans* infection in *CARD9*^−/−^ mice, suggests that the predilection for CNS infections is due to impaired recognition of fungi by microglia that in turn leads to diminished recruitment of neutrophils (Drummond et al., 2018, 2019).

CARD9 has two binding partners, MALT1 and BCL10, that are required for signal transduction (Zhong et al., 2018). The few patients reported with loss-of-function mutations in these proteins presented with combined immunodeficiency, with the implication that MALT1 and BCL10 are indispensable in the proper development of memory T cells (Torres et al., 2014; Punwani et al., 2015). These patients also had histories of mucosal candidiasis (Torres et al., 2014; Punwani et al., 2015); it is difficult to distinguish whether fungal susceptibility was due to impaired CARD9 signaling or secondary to deficits in adaptive immunity in these patients.

### Summary

*Candida* spp. cause a broad spectrum of infectious diseases, and a growing body of evidence reveals differences in host response and susceptibility across this spectrum. RVVC is a primary illness, largely due to *C. albicans*. Perturbations of innate immunity, especially involving events at the mucosa, appear to confer susceptibility to RVVC. This includes fungal recognition, IL-22 signaling, and possibly, mucosal fucosylation. Factors that influence susceptibility to candidiasis at the oral mucosa are different; in the absence of T cells, patients are highly vulnerable to OPC and OEC. There is not yet a clear role for genetic susceptibility in modifying risk of mucosal candidiasis in the setting of immunosuppression and immunodeficiency.

A number of immune processes have been implicated in genetic susceptibility to invasive candidiasis. These again include recognition of fungal ligands, although by different receptors than those implicated in RVVC. Mild impairment of neutrophil recruitment may predispose patients to disseminated candidiasis, and in the absence of neutrophils, defects in the development of adaptive immunity may also be a risk factor. Recently, unbiased approaches have uncovered diverse processes that could contribute to genetic susceptibility, such as antiviral responses and trans-regulation of cytokine production.

Rare, “idiopathic” infections by *Candida* spp. have been extensively studied. Deficits in cellular immunity predispose patients to CMC, while deficits in innate responses, especially in the ability of neutrophils to kill yeast, predispose to invasive disease. CARD9 deficiency is notable in causing both.

## Aspergillosis

*Aspergillus* spp. are filamentous fungi that are associated with the ecological niche of saprophyte. They spread by spores, which reach great density in association with soils or decomposition and readily become airborne (Williams et al., 2019). In indoor environments, *Aspergillus* spores are associated with water-damaged structures and potted plants (Mousavi et al., 2016). Even with efforts to limit airborne contaminants, it is difficult to exclude *Aspergillus* spores from healthcare environments (Oberle et al., 2015).

*Aspergillus* spp. are associated with a number of different disease states. There are more than 11 million people who suffer from fungal allergic asthma, associated with *Aspergillus* (Bongomin et al., 2017). Genetic predisposition to allergic asthma will not be discussed here; we instead focus on diseases caused by *Aspergillus* infection *per se*. There are an estimated 3 million cases of chronic pulmonary aspergillosis (CPA) worldwide (Bongomin et al., 2017). CPA is often comorbid with chronic respiratory conditions that affect the structure or function of the lungs (Bongomin et al., 2017). Invasive aspergillosis (IA) affects some 300,000 people annually (Bongomin et al., 2017). IA is typically associated with profoundly immune compromised states; still, there is growing appreciation that patients with multiple comorbid conditions, such as chronic obstructive pulmonary disease (COPD) plus diabetes, are predisposed to IA and undercounted in estimates of prevalence of this infection (Bao et al., 2017; Bongomin et al., 2017).

Further complicating an understanding of these infections is the fact that multiple *Aspergillus* species are capable of causing illness. Biological differences between these species, plus subtle differences in the immunosuppressed states of different patient populations may contribute to the relative likelihood that IA is caused by *A. fumigatus* vs. other *Aspergillus* spp.; for example, IA in neutropenic patients is predominantly caused by non-fumigatus *Aspergillus* spp., but in bone marrow transplant patients, *A. fumigatus* was the most common species (Torres et al., 2003). This distinction has clinical relevance, as the species causing non-fumigatus aspergillosis tend to have higher minimum inhibitory concentration and minimum fungicidal concentration for clinically important antifungals (Torres et al., 2003).

### Chronic Pulmonary Aspergillosis (CPA)

Studying genetic predisposition to CPA is complicated by the heterogeneity of the patient populations and presentation of illness. The clinical spectrum of CPA includes aspergilloma and chronic cavitary pulmonary aspergillosis (CCPA), and at-risk patients include those with COPD, sarcoidosis, and chronic pulmonary infections that cavitate such as tuberculosis (Bongomin et al., 2017). While these conditions may have a component of immune dysregulation, patients with these conditions are usually not immune compromised in the classic sense.

Studies in a cohort of patients in Manchester, UK, suggest that CCPA is associated with an overly-active immune response to fungi. Macrophages from CCPA patients showed greater up-regulation of cytokines at the experiment's endpoint as compared to healthy donor cells, though CCPA patient cells also had greater lag time in their response to fungal stimuli (Smith et al., 2014a). CCPA patient cells had different PRR expression profiles as compared to healthy donor cells (Smith et al., 2014a). These authors identified risk variants in genes encoding cytokines (*IL1B, IL1RN, IL15*), PRRs [*TLR1, CLEC7A* (Dectin-1)], and the genes *DENND1B, PLAT* (plasminogen activator), and *VEGFA* (Smith et al., 2014a,b). The association between CPA and hyper-inflammatory states is supported by a study of CPA patients in Shanghai, China, which found that greater levels of IL-1β are correlated with markers of advanced illness such as aspergilloma size (Zhan et al., 2018).

A study of COPD patients with CPA suggests that infection in this context may be associated with decreased recognition of fungi. A variant in the gene encoding the PRR pentraxin 3 (*PTX3*) is associated with the development of CPA in a cohort of Chinese COPD patients (He et al., 2018). This risk variant is associated with decreased pentraxin 3 in patient plasma, but this association was not found in the group with CPA alone (He et al., 2018). Variants in *PTX3* have been extensively studied in relation to invasive aspergillosis and are discussed further below.

### Invasive Aspergillosis (IA)

Immune compromise is almost essential for IA. IA is most often associated with allogeneic hematopoietic stem cell transplant (HSCT), and almost a quarter of HSCT patients develop IA in some studies (Robin et al., 2019). Patients undergoing allo-HSCT experience an initial risk at transplant, as well as a late risk associated with milder immunosuppression from graft-versus-host disease (GVHD) (Robin et al., 2019). In all cases, studying genetic risk in allo-HSCT patient populations is complicated by the presence of both donor and recipient genetics.

Mounting evidence suggests that variants associated with weaker recognition of fungi by the innate immune system are associated with greater risk of IA. Perhaps the best studied gene in this regard is pentraxin 3, a soluble PRR that recognizes *Aspergillus* conidia (Garlanda et al., 2002). *PTX3*^−/−^ mice are unable to control *Aspergillus* infection, with deficits noted in phagocyte function, as compared to wildtype mice (Garlanda et al., 2002). Multiple studies have associated *PTX3* variants that result in decreased expression with risk of IA in HSCT patients (Cunha et al., 2014; Fisher et al., 2017; Herrero-Sanchez et al., 2018). Donor *PTX3* genotype determined risk, and recipient *PTX3* genotype had no significant effect (Cunha et al., 2014; Fisher et al., 2017).

Linked *TLR4* coding variants *D299G-T399I*, which result in decreased signaling by TLR4 (Arbour et al., 2000), have also been implicated in IA risk following allo-HSCT. Risk variants are either significant only considering donor *TLR4* genotype (Bochud et al., 2008; de Boer et al., 2011), or, in one study, considering either donor or recipient *TLR4* genotype (Koldehoff et al., 2013). Several studies, including the largest study of genetic risk of IA in allo-HSCT patients, have failed to detect a significant risk of the *TLR4-D299G-T399I* genotype (Kesh et al., 2005; Grube et al., 2013; Fisher et al., 2017); at least in some studies, this may be attributable to low frequency of the risk genotype (Fisher et al., 2017). HSCT recipients with the *TLR5-Stop* variant, missense variant *TLR1-R80T*, or both missense variants *TLR1-N248S* and *TLR6-S249P* together are at increased risk of IA (Kesh et al., 2005; Grube et al., 2013). These associations seem to arise from fungal recognition by non-hematopoietic-lineage cells, as there is no significant effect on risk of IA by HSCT donor genotype at variants in *TLR1, TLR5*, and *TLR6* (Kesh et al., 2005; Grube et al., 2013). Variants in the genes *S100B* and *RAGE* also predispose allo-HSCT patients to IA by a mechanism thought to involve hyperactivity of this innate recognition pathway, which results in TLR2 blockade and TLR3 and TLR9 activation (Cunha et al., 2011; Sorci et al., 2011). Variants associated with risk were found either in HSCT donors (*S100B* and *RAGE*), or recipients (*S100B*) (Cunha et al., 2011). There are conflicting reports as to the association of *CLEC7A-Y238X* with IA risk in HSCT; two studies have correlated risk of IA with either recipient or donor carrying this variant (Cunha et al., 2010; Fisher et al., 2017), while others have failed to detect an effect (Chai et al., 2011; Herrero-Sanchez et al., 2018).

Several other innate receptor variants increase the risk of IA in the setting of HSCT. The CLR MelLec, which recognizes fungal DHN-melanin, has been implicated in IA risk upon HSCT. HSCT donor genotype at MelLec is associated with risk, while recipient genotype is not (Stappers et al., 2018). Macrophages of the risk MelLec genotype produce less pro-inflammatory cytokine in response to *A. fumigatus* conidia than do cells of the alternate genotype (Stappers et al., 2018). In contrast, IA rates are higher in HSCT donors that harbor a pro-inflammatory variant of *NOD2*, an intracellular PRR, whereas genetic deficiency of *NOD2* confers resistance to IA (Gresnigt et al., 2018). While not innate fungal recognition *per se*, a coding variant in plasminogen has been implicated in IA risk; the risk is significant when considering HSCT recipient genotype and not donor genotype, which is consistent with the hepatic origin of plasminogen (Zaas et al., 2008). The authors suggest that differences in interaction between plasminogen and *Aspergillus* conidia could lead to differences in local inflammation and pathogen entry (Zaas et al., 2008). Considered intracellular signaling downstream of PRRs, one study of variants in multiple NF-κB-related genes failed to uncover significant associations with risk of IA in HSCT patients, although there may have been a weak association with a haplotype at *IRF4* (Lupianez et al., 2016).

Multiple variants associated with risk of IA following HSCT have been identified in genes involved in the development of appropriate T cell responses. A haplotype in *CXCL10* is associated with risk of IA following HSCT (Mezger et al., 2008). Cells with the risk haplotype were found to produce less CXCL10 upon stimulation with *A. fumigatus* germlings than cells with the alternate haplotype (Mezger et al., 2008). One role of CXCL10 is recruitment of Th1 cells, thus the risk haplotype may be associated with a relatively weaker Th1 response. The AspBIOmics Consortium uncovered several IA risk variants that could be associated with altered T cell responses; these associations were stronger in HSCT patients than in the larger pool of at-risk hematology patients (Lupianez et al., 2016). Risk variants were identified in *IL4R, IL8, IL12B*, and *IFNG*, with experiments demonstrating functional effects of the *IFNG* variant on IFNγ production and related endpoints (Lupianez et al., 2016). A risk variant that increases IL-10 production would suppress T cell responses. Donor genotype at a variant upstream of *IL10* is associated with development of IA, and the risk genotype leads to increased production of the anti-inflammatory cytokine IL-10 (Cunha et al., 2017). Mouse models of *Aspergillus* infection support this finding; *IL10*^−/−^ animals are more resistant to experimental aspergillosis (Clemons et al., 2000). Taken together, these findings suggest that variants that lead to relatively weaker Th1 responses may predispose HSCT patients to IA.

Much of what is known about genetic predisposition to IA following HSCT has been reiterated in studies of other vulnerable populations. *PTX3* variants are associated with risk of IA in patients with COPD (He et al., 2018), or following solid organ transplant (Wojtowicz et al., 2015b). A small study of patients with hematologic malignancies found that expression of *S100B* was significantly associated with IA (Dix et al., 2016). Three studies have demonstrated that *CLEC7A* (Dectin-1) or *CD209* (DC-SIGN) variants are associated with the development of IA in patients with hematologic malignancies (Oberhofer, 1979; Sainz et al., 2010b, 2012), and this is further supported by a study finding decreased *CLEC7A* expression in hematologic malignancy patients with IA (Camargo et al., 2015). While genetic studies have not been done, MBL levels are lower in immunocompromised patients with IA vs. febrile immunocompromised controls (Lambourne et al., 2009). As discussed in the section on candidiasis, loss-of-function MBL variants predispose patients to RVVC, but no association has been found for candidemia. The risk associated with relatively greater production of IL-10 was reiterated in a study of patients with hematologic malignancies, including some that had undergone HSCT (Sainz et al., 2007a).

Recent work has also addressed the role of early responses, especially cytokine signaling, in predisposition to IA in high-risk populations aside from HSCT patients. Variants impacting IL-1β are reported to influence risk of IA in patients with hematologic malignancies, and following solid organ transplant; the genes harboring risk variants include *IL1A, IL1B, IL1RA*, and *IL1RN* (Sainz et al., 2008; Wojtowicz et al., 2015a). When stimulated with *A. fumigatus* conidia, cells harboring these variants produce less of several cytokines, including IL-1β and TNF-α, than do control cells (Wojtowicz et al., 2015a). Signaling through TNF has also been implicated in two studies of patients with hematologic malignancies, some of whom underwent HSCT; these studies report significantly higher rates of IA in patients with genetic variants that decrease expression of *TNFR1* and *TNFR2* (Sainz et al., 2007b, 2010a; Li and Anderson, 2018). One report also identified a variant in the promoter of *DEFB1*, encoding β-defensin 1, associated with IA in solid organ transplant patients (Wojtowicz et al., 2015a). As with IL-1β and TNF-α, β-defensin 1 helps coordinate the early immune response by acting as a chemotactic signal. Direct anti-*Aspergillus* activity of β-defensin 1 has not been investigated.

### “Idiopathic” Aspergillosis

*Aspergillus* infection in otherwise healthy people is rare, and generally associated with monogenic susceptibility. The best example of this is *Aspergillus* infections in patients with ineffective neutrophil oxidative bursts due to CGD; these patients are at risk for both pulmonary and disseminated aspergillosis (Wolach et al., 2017). CARD9 deficiency has also been identified in patients who present with extrapulmonary *Aspergillus* infection without evidence of pulmonary aspergillosis (Rieber et al., 2016). HIES, which is STAT3 deficiency, is associated with susceptibility to invasive aspergillosis (Vinh et al., 2010; Dureault et al., 2019). Interestingly, CARD9*-* and STAT3-deficient patients are similar to control donors in terms of neutrophil responses to *A. fumigatus* (Vinh et al., 2010; Rieber et al., 2016).

It is not clear whether STAT3 deficiency alone is sufficient to predispose patients to aspergillosis. HIES patients develop aspergillosis later in life, after suffering other structural insults to the lung (Dureault et al., 2019). Extrapulmonary aspergillosis is reported, however, in association with STAT3 haploinsufficiency that did not appear to impact antifungal Th17 responses (Natarajan et al., 2018). STAT3 is pleiotropic, and dozens of mutations in *STAT3* are associated with varied impacts on the final gene product (Vogel et al., 2015); future reports may clarify the spectrum of disease that results from select mutations.

### Summary

Aspergillosis encompasses varied clinical manifestations, and the differences are manifest in the genetic predisposition to different forms of *Aspergillus* infection; from hyper-inflammatory responses that predispose to CPA to ineffective fungal recognition that predispose to IA. Despite the difficulty of studying these varied illnesses, progress has been made toward identifying and validating risk variants, especially in the context of HSCT. In the coming years, this avenue of research holds great promise for improving HSCT outcomes, both by informing donor selection and by proactively identifying those recipients at the highest risk of IA.

## Endemic Dimorphic Fungal Infections

Dimorphic fungal pathogens that are native to North America, including the genera *Histoplasma, Coccidioides*, and *Blastomyces*, infect hundreds of thousands of people per year (Bongomin et al., 2017). Yet, the majority of these infections are asymptomatic. Puzzlingly, many of those who develop symptoms do not appear to be at increased risk for fungal infection; they are young and seemingly healthy. Additionally, each of these fungal infections has a higher incidence in select groups, especially people of African, Native American, or Asian ancestry. On the basis of these two observations, a significant role for host genetics has long been supposed for these infections.

### Histoplasmosis

Histoplasmosis is the most prevalent of the dimorphic fungal infections, with an estimated 500,000 infections per year (Bongomin et al., 2017). As with the other dimorphic fungi, *Histoplasma capsulatum* grows in organic soils; outbreaks have been associated with disturbances that lead to aerosolization of infectious spores, e.g., construction in both rural and urban settings (Deepe, 2018). *H. capsulatum* is also associated with bird and bat droppings; thus, outbreaks have been associated with clearing bird waste and disturbing bat guano in caves and tunnels (Deepe, 2018). Although the geographic distribution of *H. capsulatum* is often quoted to be the Ohio River Valley, suitable environmental conditions exist to support the fungus throughout the upper Midwest (Maiga et al., 2018).

The vast majority of exposures to *H. capsulatum* do not result in clinical illness. In fact, virtually the entire population in some highly endemic areas may be repeatedly exposed (Goodwin et al., 1981). Pulmonary nodules are a common incidental finding on medical imaging of patients living in endemic areas; in highly endemic areas, up to 12% of these nodules may be associated with infection with *H. capsulatum* (Benedict et al., 2019; Deppen et al., 2019). Such latent infections are usually asymptomatic, although they may present as invasive disease decades later upon immunosuppression (Bourgeois et al., 2011). In immunocompetent individuals, histoplasmosis most commonly manifests as isolated fungal pneumonia (Ouellette et al., 2018), and acute illness is often associated with high levels of exposure to infectious spores (Deepe, 2018). There is a noted racial health disparity, with historical reports of higher incidences of histoplasmosis amongst black people in outbreaks (Wheat et al., 1981); however, these reports may be confounded by geographical factors.

Histoplasmosis poses a threat to organ transplant recipients (Tanveer et al., 2019) and patients with AIDS (Assi et al., 2006; Wheat et al., 2018). Disseminated histoplasmosis is most commonly associated with the immunocompromised patient, and rare in immunocompetent patients (Ouellette et al., 2018). Sites of dissemination include the central nervous system (Wheat et al., 2018) and gastrointestinal tract (Assi et al., 2006). Another recognized risk of disseminated histoplasmosis is TNF-α blockade, for example in the treatment of inflammatory bowel disease (Smith and Kauffman, 2009; Vergidis et al., 2015). As with overall risk of infection, black race has been associated with increased risk of severe histoplasmosis in AIDS patients (Wheat et al., 2000). There are currently no reports addressing genetic determinants of racial disparities in histoplasmosis pneumonia or disseminated histoplasmosis.

Mouse models of histoplasmosis have recapitulated many of the features of human susceptibility. TNF-α is required for the establishment of memory and control of infection (Zhou et al., 1998; Deepe, 2006). Type 1 cytokines are indispensable to control of infection (Allendoerfer and Deepe, 1997), and Th17 cells contribute to protective immunity (Deepe et al., 2018). However, IL-17 responses are ultimately not required for survival of infection in the mouse model (Deepe and Gibbons, 2009). Th2 responses prolong illness, perhaps by delaying the development protective Th1 cells (Gildea et al., 2003).

Case reports concerning patients with mutations that affect immunity further elucidate the immune responses that are required for control of *H. capsulatum*. Patients with deficient type 1 cytokine responses are at risk for disseminated histoplasmosis; such deficiencies include loss-of-function mutations impacting IFNγR1 (Zerbe and Holland, 2005) and IL12Rβ1 (Rosain et al., 2018). Histoplasmosis is reported in patients with HIES resulting from loss-of-function mutations in STAT3 (Robinson et al., 2011; Odio et al., 2015), as well as gain-of-function mutations in STAT1 (Sampaio et al., 2013); this could be related to impaired Th17 responses, other perterbations resulting from loss of these transcription factors, or a combination of Th17-dependent and Th17-independent factors. Patients lacking NEMO, a component of NF-κB signaling (Lovell et al., 2016), or GATA2, a transcription factor involved in the differentiation of myeloid cells (Collin et al., 2015) are also very susceptible to disseminated histoplasmosis. This could be due in part to impaired macrophage and monocyte responses, including signaling downstream of TNF-α; however, loss of NEMO or GATA2 result in very complicated immune disturbances, so it is not possible to attribute this susceptibility to any one mechanism.

### Coccidioidomycosis

While coccidioidomycosis is a clinical entity similar to histoplasmosis and blastomycosis, the genus *Coccidioides* is more closely related to the causative agents of dermatophytosis than to other dimorphic fungal pathogens (White et al., 2014; Whiston and Taylor, 2015). This may account for subtle differences in environmental niche and antifungal immunity against *Coccidioides* spp., compared to *Histoplasma* spp., and *Blastomyces* spp. Coccidioidomycosis is mainly reported in the southwestern United States, and is associated with a complex set of climactic conditions including alternating wet and dry periods (Shriber et al., 2017).

There are an estimated 25,000 cases of coccidioidomycosis that require medical treatment per year (Bongomin et al., 2017), with an incidence of more than 40 cases per year per 100,000 population in endemic states (Centers for Disease Control Prevention, 2013). Up to 29% of community-acquired pneumonia may be due to *Coccidioides* spp. in highly endemic areas (Valdivia et al., 2006). Still, a minority of those who are exposed to *Coccidioides* spp. develop acute illness; the seropositivity rate to the fungus is about 30% in endemic areas (Dodge et al., 1985), with reports of over half of the population being reactive to *Coccidioides* antigen in some areas (Nguyen et al., 2013). Chronic infection is common, with up to 29% of lung nodules being attributable to *Coccidioides* spp. in endemic areas (Forseth et al., 1986).

Progression to disseminated coccidioidomycosis is rare, occurring in fewer than 1% of infections (Stevens, 1995). Immunosuppression increases risk of dissemination, as does use of TNF-α inhibitors (Bergstrom et al., 2004; Blair et al., 2019). It has long been recognized that the risk of dissemination is not equal across people of different races and ethnicities. Native American (McCotter et al., 2019), African American (Ruddy et al., 2011), and Pacific Islander (Drake and Adam, 2009) ancestry all confer increased risk of dissemination.

In mouse models of coccidioidomycosis, recognition of the fungus by Dectin-1 is required to control infection (Viriyakosol et al., 2013; Feriotti et al., 2015), as is downstream signaling by CARD9 (Hung et al., 2014). MyD88 is also required for the induction of protective T cell responses (Hung et al., 2014; Viriyakosol et al., 2018). Considering the critical role of MyD88, it is surprising that TLR recognition of fungal ligands is largely dispensable; instead, the role of MyD88 may reflect signaling downstream of the IL-1β receptor (Hung et al., 2014; Viriyakosol et al., 2018). Th1 and Th17 responses contribute to control of infection (Hung et al., 2011). While Th1 responses are dispensable, Th17 responses are not (Hung et al., 2011).

Many of the same genetic deficits in immunity that predispose patients to disseminated histoplasmosis also confer risk of disseminated coccidioidomycosis. There are case reports to this effect for loss-of-function mutations impacting IFNγR1 (Vinh et al., 2009), IL12Rβ1 (Vinh et al., 2011), STAT3 (Odio et al., 2015), and gain-of-function in STAT1 (Sampaio et al., 2013). Interestingly, based on a small number of cases reported to date, it appears that the fungus may disseminate preferentially to distinct sites depending on which component of immunity is impaired. STAT3 mutations are associated with dissemination to the CNS, while mutations that more directly impact Th1 responses are associated with dissemination to bone and lymph node (Odio et al., 2015).

A few reports have addressed population-level patterns of susceptibility to disseminated coccidioidomycosis. Some of the risk may be attributable to differences in antigen presentation, as certain HLA alleles are associated with dissemination in specific populations (Louie et al., 1999). More recently, a preliminary description of a genomic study of 58 patients with disseminated coccidioidomycosis identified 103 rare variants in 21 genes associated with antifungal immunity (Hung et al., 2019). Affected pathways included IL-17 signaling, IL-12-IFN-γ signaling, and NF-κB signaling (Hung et al., 2019). Two thirds of patients had functionally relevant variants with a population frequency of <0.1%, including several patients with biallelic deleterious variants in key genes (Hung et al., 2019).

### Blastomycosis

The ecology of *Blastomyces* spp. is somewhat enigmatic, due largely to difficulty in isolating the fungus from the environment (Reed et al., 2008). The microbial physiology of *Blastomyces* spp. suggests they are adapted for woody substrates and possibly animal waste (Baumgardner and Laundre, 2001). *Blastomyces dermatitidis* and the closely related *B. gilchristii* are classically associated with waterways (Reed et al., 2008; McTaggart et al., 2016). The two species have somewhat different distributions, with *B. dermatitidis* found throughout eastern North America and *B. gilchristii* largely restricted to Canada (McTaggart et al., 2016). *Blastomyces* strains isolated from patients cluster by clinical presentation, suggesting a contribution of fungal genetics to clinical outcomes (Meece et al., 2013).

Most cases of blastomycosis are associated with outdoor activities involving exposure to soil (Klein et al., 1986; Choptiany et al., 2009). While outdoor recreation or such activities as forestry are a common source of exposure, blastomycosis is also reported from the urban setting (Pfister et al., 2011). Periodic outbreaks of blastomycosis are associated with a point source where multiple people are exposed to the infectious spores (Pfister et al., 2011); multifocal outbreaks have also been described, and may be associated with environmental factors that produce ideal conditions for sporulation or aerosolization of spores (Roy et al., 2013). As with the other dimorphic fungal infections, evidence suggests that exposure to *Blastomyces* spp. is more common than appreciated in endemic areas, and that the majority of these exposures do not lead to clinical presentation (Vaaler et al., 1990).

The majority of blastomycosis cases present as fungal pneumonia (Baumgardner et al., 1992; Castillo et al., 2016). Though blastomycosis is comparatively uncommon, with perhaps only 3000 cases reported annually (Bongomin et al., 2017), in highly endemic areas, the incidence may reach 40 cases per year per 100,000 population at risk (Baumgardner and Brockman, 1998). The majority of cases are in people under the age of 50 who are immunocompetent (Baumgardner et al., 1992). Blastomycosis is more common in people of African American, Native American, and Asian, especially Hmong, ancestry (Howard, 1984; Baumgardner et al., 2002; Roy et al., 2013; Khuu et al., 2014). During outbreaks, the incidence of blastomycosis in these select populations is reported to be as high as 277 cases per year per 100,000 populaton at risk (Baumgardner et al., 2002). Some of these associations may be due to a combination of genetic and non-genetic factors. However, comorbidity and exposure risk were recently excluded as contributors to a greatly increased incidence rate of blastomycosis during investigation of a large Wisconsin outbreak that included Asian people, strongly implying a genetic risk (Roy et al., 2013).

There is substantial heterogeneity in the clinical presentation of blastomycosis. A minority of patients present with severe pneumonia, even Acute Respiratory Distress Syndrome (ARDS); this is thought to be a function of exposure to an especially large inoculum in many of the cases (Castillo et al., 2016). From 15 to 40% of patients develop blastomycosis outside of the lung, with the most common site of dissemination being the skin (Baumgardner et al., 1992; Castillo et al., 2016). Immunosuppression is a risk factor for both ARDS and dissemination, although there is not an apparent, overall increased risk of infection (Choptiany et al., 2009; Castillo et al., 2016). Limited data suggest that patients on TNF-α inhibitors may be more susceptible to infection (Castillo et al., 2016).

Studies in a mouse model of blastomycosis have revealed a critical role for early responses by lung epithelial cells (Hernandez-Santos et al., 2018). MyD88 is indispensable in the response to *B. dermatitidis*; *MyD88*^−/−^ mice succumb to lethal infection even by an attenuated vaccine strain that is non-lethal in wild-type mice (Wang et al., 2016). Signaling through IL-1R1 drives protective Th17 immunity (Wuthrich et al., 2013; Wang et al., 2014; Hernandez-Santos et al., 2018). Interestingly, Tc17 cells confer durable protection in CD4-deficient animals (Nanjappa et al., 2012). Innate, IL-17-producing lymphocytes also play an important role (Hernandez-Santos et al., 2018). As with the other dimorphic fungal infections, TNF-α coordinates antifungal immunity to blastomycosis (Finkel-Jimenez et al., 2001).

Recently, work has addressed the genetic underpinning of health disparities in rates of blastomycosis in otherwise healthy individuals. A homozygosity mapping approach was used to study 9 Hmong blastomycosis patient genomes, and candidate susceptibility variants were identified on the basis of their rarity in European populations and other features that may indicate that a given variant influences susceptibility (Merkhofer et al., 2019). This approach revealed 113 candidate susceptibility variants. The authors validated the impact of a block of variants near IL6 that is nearly fixed in Wisconsin Hmong at large but rare in European populations (Merkhofer et al., 2019). Among other readouts, the authors found that healthy Wisconsin Hmong donors had relatively hypoactive IL-6 and antifungal Th17 responses compared to healthy European donors (Merkhofer et al., 2019). The latter finding is consistent with differences in IL-6 responses, which could also explain several as-yet unstudied population differences that impact T cell development. To date, the only reported monogenic condition associated with blastomycosis is GATA2 deficiency (Spinner et al., 2016).

### Summary

A genetic basis for susceptibility to endemic dimorphic fungi has long been hypothesized, due to their propensity for causing disease in otherwise healthy people, and to their predilection for people of certain genetic backgrounds. Extensive experiments in mouse models have highlighted similarities and differences in the immune responses to this diverse group of pathogens. Only in the past few years have studies in patients begun to reveal those processes that are critical to human immunity. The role of Th1 and Th17 responses is evinced by the severe infections seen in patients with monogenic deficits in those pathways. More recently, studies of disseminated coccidioidomycosis and pulmonary blastomycosis have revealed subtle variants in these pathways. The identification of these variants represents progress toward understanding the genetic underpinnings of observed health disparities in these pathogens.

## Applying What is Understood of Genetic Susceptibility to Fungal Diseases

While an increasing number of mutations and variants are reported to confer risk of fungal diseases, only PIDs and Mendelian susceptibility variants are currently clinically actionable. This is partially because there are substantial challenges in translating these findings to clinical applications. By necessity, most studies of susceptibility to fungal diseases have limited their donor population to one ethnic/racial background, which may limit generalizability of findings or, at the very least, necessitate extensive validation of variants in diverse patient populations. Still, the availability of next-gen sequencing technologies may soon make it feasible to incorporate testing for subtle risk variants into clinical practice, and guidelines will be developed for how to incorporate an individual's susceptibility profile into patient care. Future efforts may seek to develop tools to stratify patients by risk based on susceptibility variants; for example, IA susceptibility variants could be incorporated into a tool to stratifying potential HSCT donors by risk of invasive molds.

## Author Contributions

RM and BK conceived the content of the manuscript together. RM drafted much of the manuscript. BK contributed to portions, and reviewed and edited the entire manuscript. RM and BK jointly finalized the manuscript.

### Conflict of Interest

The authors declare that the research was conducted in the absence of any commercial or financial relationships that could be construed as a potential conflict of interest.
